# The Institutional Worker

**Published:** 1920-02-14

**Authors:** 


THE
### INSTITUTIONAL WORKER
**Being a Special Supplement to "The Hospital"**

### OUR BUREAU OF INFORMATION.

### Rules for Correspondents.

1. Every letter must be accompanied by the coupon to be cut from the back cover (inside page) of THE HOSPITAL, current issue, and must contain the name and address of the correspondent with pseudonym for publication if desired. Replies by post cannot be given save under exceptional circumstances at the Editor's discretion.

2. Letters from Approved Homes in reply to special needs published in the Bureau should state terms and full particulars, and be sent prepaid under cover to the Editor of the Bureau with name written across coupon for identification.

3. Proprietors of Homes which have not yet been entered on the List of Approved Homes, but have spare accommodation likely to suit special needs, are invited to write for an application form for registration. The fee for registration, which includes two announcements of the Home in the Bureau and other privileges, is 10s.

4. All communications to be addressed to the Editor of THE HOSPITAL, 28 Southampton Street, Strand, London, W.C. 2, and marked " Bureau of Information."

### INSTITUTION FACTS AND FIGURES.

### The Question Box.

#### THE UTILISATION OF VOLUNTARY HELP.

THE question was :

**How would you deal with offers of voluntary part-time help in connection with your hospital from professional men, clerks, &c.? Give suggestions how their assistance might be utilised.**

**" G. W." replies:**

In many ways voluntary assistance is profitable to a hospital, but it should also be borne in mind that there is danger in allowing the outside world to engage in the more confidential management of the institution. Conflicting unpaid minds are liable to cause much friction if not discreetly used. Voluntary workers sometimes disagree with the policy of the board of management, and in consequence the hospital loses their services, and sometimes their financial assistance. By confining their services to outside efforts this is avoided.

The offers of voluntary help should be classified, and interviews arranged with the house governor and secretary. The capabilities of the volunteers should be tested, for we cannot afford to have our work done by those who are not fitted for the positions they desire to fill. It would be left to the house governor and secretary diplomatically to refuse help not desirable, for he knows the requirements of his own hospital. The organisation of whist-drives, dances, and concerts can very well be done by voluntary professional men who are well known in the immediate district. The hospital officers have not time to devote to these things, being engaged in more intimate work. The name of a local professional man goes a long way to advertise such an effort.

Much good work can be done by professional men in their own business by a strong personal appeal to their employees to recognise the hospital financial crisis and to increase their contributions to the local institution. Any assistance they require could be given by hospital officers.

In reference to the clerical help, this could be employed for entering up the in-patient and out-patient registers, but beyond this it is not advisable to go.

Voluntary help from the medical and surgical professions is already accepted in the majority of institutions, so it is not necessary for me to comment further upon it.

The motor ambulances of our institutions could be driven by voluntary men if there were any available living in close proximity to the hospital. This would save much expense, for a good driver requires a good wage. If there happened to be an architect among the volunteers it would be a gain to consult him when reconstruction is in progress.

A great amount of voluntary help can be given by the hospital Saturday Committee when there are tickets to be disposed of in connection with concerts, whist-drives, etc. This method must be used discreetly so as not to weary the representatives.

Ladies can help by the making of garments for the use of patients during their period in hospital, and if a sufficient amount of help is forthcoming a special committee could be formed to deal with the purchase of materials, etc.

**"Lancastrian" replies:**

Offers of voluntary part-time help should be welcomed broadly from three aspects : relieving the pressure of work which to-day especially falls upon a hospital secretary and his staff ; increasing scope for larger activities ; creating greater interest in the institution's affairs. Clerical services can be most useful in the work of appeal—*e.g.*, new subscribers, house-to-house collections, fêtes, dances, whist-drives, garden parties, concerts, Hospital Saturday and Sunday Funds, etc.—and in coping with arrears of work, such as account-books, statistical data, etc.

Here assuredly help can be of inestimable value in easing the secretary's burden and in obtaining further financial support.

Legal assistance in connection with legacies, property, rents, relationship of employer and employed in matters of salaries and wages, serving of notice, agreements, etc., contracts, schemes (Venereal Disease, Pensioner, etc.) watching the interests of the hospital in respect of inquests, and in not a few other ways. This, too, is most helpful service.

Then again, how valuable is honest and wise advice from skilled and experienced chemists ! What economies and improvements can oft-times be effected by such in a hospital dispensary ! Not a few institutions must have been saved considerable expenditure during the war, especially by having the guidance of one or more honorary pharmacists.

An electrical engineer can be an undoubted asset to a hospital. There is to be an improved electric-lighting scheme, or a department which is without such illumination is to have it installed, or some electro-therapeutic apparatus requires overhauling—in the preparation of specifications, the question of the acceptance of tenders, etc., can much good work be accomplished.

And last, but not least, is the invaluable co-operation of ladies—*e.g.*, in augmenting funds and in obtaining linen, etc., and making garments.

### Mental Nursing in South Africa and New Zealand.

WE suggest that you write to the Secretary of the Medico-Psychological Association, 11 Chandos Street, London, W., for advice upon Mental Nursing in South Africa and New Zealand. A coupon should be enclosed with each inquiry. We cannot give postal replies.—D. M. N.

### Question for February.

### A NURSE'S VENTURE AS OPTICIAN.

**A trained nurse with long experience in an eye hospital who has taken the certificate of the British Optical Association, is desirous of setting up business in a flourishing provincial town. Give suggestions and advice as to size of shop, its fittings, capital required, staff required, and the best method of making the shop known by advertising or otherwise.**

**A minimum payment of 10s. 6d. will be made for each published answer.)**

RULES.

The following rules must be observed:—

1. Contributions must be written on one side of the paper. Conciseness and terseness are desirable features. The MS. must bear the name and address of the sender and be accompanied by coupon to be cut from the back cover (inside page) of the current issue of THE HOSPITAL. A pseudonym must be chosen if the name is not to be published.

2. Contributions must be addressed to the Editor of THE HOSPITAL Institutional Worker Supplement, 28 & 29 Southampton Street, Strand, London, W.C. 2, should reach him before the end of the current month, and be marked in the left-hand corner "Facts and Figures."

### QUESTIONS INVITED.

IN connection with our Question Box, we offer every month five shillings each for the best questions which are sent in for consideration. The questions may be on any institutional subject and concern any department, from the secretary's to the porter's, or the matron's to the domestic staff; the one essential is that they must be practical and deal with points that have a definite relation to hospital or institutional administration, upkeep, finance, or management. Points relating to artificers' work, housekeeping, the laundry, out-patients, and other practical matters will be welcome. It is hoped that thus, when difficult or doubtful points arise in the course of their work, institutional workers will be encouraged to put them in the form of a question and send them to the Editor, THE HOSPITAL Bureau of Information, 28 & 29 Southampton Street, Strand, London, W.C. 2, marked "Question Box."

The best questions will be published in due course, and our readers will be given the opportunity of answering them. Thus all institutional workers may be able to cooperate with the view of helping the work and smoothing the difficulties of each other. In short, we want the Question Box of the *Institutional Worker* to be freely used by every worker habitually.

### ENQUIRIES AND ANSWERS.

### *EMPLOYMENT AND TRAINING.*

### Massage Training.

IT is not essential to enter a London hospital to gain your massage certificate; it is possible to obtain the necessary training through a private teacher, the student attending a hospital for practical work for a certain number of hours during the twelve months' course of training. We suggest that you write to the Secretary of the Incorporated Society of Trained Masseuses, 157 Great Portland Street, London, W. 1, who will send you a list of their training-schools and private teachers. A coupon should be enclosed with each inquiry. We cannot give postal replies. —D. M. C.

### Training in Special Work Before Entering General Hospital.

DOES the nursing of children appeal to you? You could gain very useful experience in a children's hospital before going in for training in general nursing. Write to the Matrons of the following institutions:—Hospital for Sick Children, Great Ormond Street, London, W.C.; Infants' Hospital, Vincent Square, Westminster, London, S.W.; and Lord Mayor Treloar Hospital for Crippled Children, Alton, Hants. You might obtain "How to Become a Nurse," published by The Scientific Press, Ltd., 28 and 29 Southampton Street, Strand, W.C. 2, which contains a full list of training-schools and much other useful information for those intending to take up nursing. The price is 2s. 10d. post free. We regret we cannot give postal replies. A coupon should be enclosed with each inquiry.—H. D.

### Nursing in New Zealand.

WITH reference to our paragraph in last week's issue under this head, we are informed that applications should be addressed to the High Commissioner for New Zealand, 415 Strand, W.C. 2, and not to the Agent-General.

### Australian Trained Nurses' Association.

THE address of the above is now The B.M.A. Building, 30 Elizabeth Street, Sydney, where the Association removed from the Equitable Building some years ago. Miss M. E. Gerran, who was Secretary of that Association and of the New South Wales Bush Nursing Association, has given up the former office, and now only retains the secretaryship of the Bush Nursing Association, whose office is transferred to Twyford House, 17 Castlereagh Street, Sydney, N.S.W.

**The Editor will be glad to receive correspondence and to consider contributions upon all subjects relating to institutional work which affect the welfare of institutional workers.**

### *THE SICK AND IN NEED.*

### Home for Old Lady.

THERE are two homes in the region suggested that might possibly suit the old lady referred to. They are the Alexandra Home for Chronic Invalids, St. Peters Road, St. Leonards-on-Sea, where patients are admitted for a payment of 10s. weekly and upwards, and the Home of the Holy Rood, Worthing; the charges range from 12s. 6d. to £3 3s. per week.—DEAL.

### Edith Cavell Homes.

THE address you ask for is "Edith Cavell Homes," 35 Victoria Street, London, S.W. 1. The new home is at Crossways, Windermere.—REST.

### *MISCELLANEOUS.*

### Grants of Red Cross Funds to Metropolitan Hospitals.

GRANTS from the surplus funds of the British Red Cross Society will be distributed to the Metropolitan Institutions only through King Edward's Hospital Fund for London. The sum of £250,000 was allocated by the British Red Cross Society for the purpose. Grants will be made in aid only of improvements and extensions. A special form of application was issued by King Edward's Hospital Fund on January 23 last, and if you have not received one you should write to the Secretary for a copy.—PERPLEXED.

### Laboratory Workers' Association.

WE regret we do not know of any association such as you mention.—W. W. S.

### *PRACTICAL MATTERS.*

### Concrete or Terazzo Floors.

As your floor has only recently been laid it is inadvisable to do more than fill the cracks with cement coloured to match the floor. In two or three years, when no further settlement is likely to occur, the cracks can be cut out and properly treated. Possibly your floor has not been well finished; if the surface is not good you can have it rubbed down. Your builders would get this done. We do not advocate the use of Ronuk on a terazzo floor, nor is it good to use soap when cleaning without the addition of a scouring powder. "Gospo" is one of the best preparations we know.—F. E. L.

### Special Covering for Water or Steam Pipes and Flanges.

AN excellent removable covering for pipes and flanges, known as "Viceroy," is also made by Bell's Asbestos Company, Ltd. It is made in sections to fit flange and pipe, and its construction in air-cell formation adds to the non-conducting properties of the asbestos fibre. Asbestos rope lagging for pipes can also be obtained.—M. T.

